# Imaging In Acute Bronchiolitis: Evaluation of The Current Practice In a Kuwaiti Governmental Hospital and Its Possible Impact on Hospitalization Period

**DOI:** 10.2174/1874306401812010075

**Published:** 2018-11-14

**Authors:** Alaa Abdel-Kader, May Fouad Nassar, Zahra Qabazard, Mohamed Disawi

**Affiliations:** 1 Pediatric Department, Al-Adan Hospital, MOH, Kuwait; 2 Pediatric Department, Faculty of Medicine, Mansoura University, Dakahlia, Egypt; 3 Pediatric Department, Faculty of Medicine, Ain Shams University, Cairo, Egypt

**Keywords:** Bronchiolitis, Imaging, Kuwait, Hospitalization, Period, X-ray, Pediatricians

## Abstract

**Background and objectives:**

Guidelines for acute bronchiolitis recommend primarily supportive care, but unnecessary treatment measures remain well documented. This study was designed to assess the Al-Adan Hospital pediatricians` attitude towards imaging of inpatients with bronchiolitis aiming to evaluate its utilization and possible impact on patients` management and length of hospital stay.

**Subjects and methods:**

This study included 194 cases of acute bronchiolitis admitted to Al-Adan Hospital. Number of X-Rays done following admission and reasons stated in the files were recorded. Bronchiolitis severity was estimated from the data obtained.

**Results:**

Chest X-Rays were ordered in 52.1% of our inpatients with acute bronchiolitis. In nearly half of those cases, the reason for X-Ray request is a clinical severity factor, namely desaturations and apneas, and in rest of the cases, no specific reason for ordering X-Rays was documented. Significantly more patients who had two or more X-Rays were prescribed antibiotics and had statistically longer hospital stay. The number of X-Rays performed during admission was not a significant contributor to the need for PICU care, however, it was a significant factor affecting the length of hospital stay.

**Conclusion:**

The implementation of acute bronchiolitis guidelines regarding imaging in admitted cases with acute bronchiolitis is highly recommended in Al-Adan hospital. Clear documentation for the reasons behind ordering X-Rays is needed for those cases. A decrease in the X-Ray utilization and subsequent unnecessary antibiotic use can help in decreasing the costs and hazards of hospitalization for patients with acute bronchiolitis.

## 1 INTRODUCTION

Although evidence-based guidelines for acute viral bronchiolitis recommend primarily supportive care, unnecessary treatment measures are still practiced [1]. Arnoux *et al.* [2], recommended that a chest X-ray is only indicated when the clinical course is unusual or if a differential diagnosis is suspected.

Bronchiolitis can be diagnosed on the basis of clinical signs and symptoms in a young child with wheezing and hyperinflation. Chest radiographs are generally unhelpful and not required in children with a clear clinical diagnosis of bronchiolitis [3]. This was previously emphasized by the American Academy of Pediatrics [4], in their guidelines which mentioned that when clinicians diagnose bronchiolitis on the basis of history and physical examination, radiographic or laboratory studies should not be obtained routinely.

In children with typical bronchiolitis, X-rays rarely produce unexpected findings. However, the abnormalities compatible with uncomplicated bronchiolitis often prompt physicians to prescribe antibiotics thus the recommendation of Suchuh *et al.* [5], is suggested against chest imaging in infants with typical bronchiolitis, as a means to reduce unnecessary antibiotic use.

A multicenter collaborative to reduce unnecessary care in inpatient bronchiolitis reported significant decline (44%) in chest radiography use upon using pediatrics network quality collaborative for improving hospital compliance with AAP bronchiolitis guideline [1].

Since data from Kuwait is scarce in this matter, the current pilot study was designed to assess the Adan Hospital pediatricians` attitude towards imaging of inpatient cases of acute bronchiolitis aiming to evaluate its utilization and possible impact on patients` length of hospital stay and antibiotic prescriptions.

## 2 MATERIALS AND METHODS

This retrospective analytical study included 194 cases of moderate to severe bronchiolitis admitted to the Pediatric Department wards in Al-Adan Hospital (located in Al-Ahmadi Governorate), Ministry of Health, Kuwait. The study was conducted between October 2015 and May 2016 and included all patients admitted with moderate to severe acute bronchiolitis and had none of the exclusion criteria. Patients’ files were reviewed and data relevant to the study were obtained and processed thus the ethical research committee approval was waived.

The performance of chest X-ray before ward admission if any, was documented. The number of X rays done following admission to the pediatric wards and reasons stated in the files were recorded. The patients were then divided into three groups; control group are those patients with no X-ray required during their admission for acute bronchiolitis. Group one are those cases who had only one X ray done during their admission. Finally, group two are those who had two or more X rays done.

The following data were collected; age, sex, poor feeding, activity, retractions, respiratory rate, grunting, wheezing, temperature on admission, lowest O_2_ saturation, lowest pH, cyanosis, development of significant apnea. Clinical data used to score bronchiolitis based on the severity of signs and symptoms according to Liu *et al.* [6], were recorded on admission, worst recorded value, and at times for X-ray request if any. Length of ward stay, Pediatric Intensive Care (PICU) admissions were documented. Although viral PCR studies were not mandatory for the diagnosis of bronchiolitis, the results were documented if available.

Exclusion criteria included history of prematurity, congenital heart disease, chronic lung disease, major congenital malformations, immune deficiency, cases receiving antibiotic for possible sepsis/pneumonia, previous admission with bronchiolitis or wheezy chest, cases with gastro-esophageal reflux disease, and incomplete medical records with the inability to retrieve required data.

IBM SPSS Statistical package for social science, version 20, was used for data analysis. Descriptive statistics were generated for demographic factors, chi-square test and fisher’s exact test were used to compare categorical data and t-test was used to compare continuous data. For the assessment of possible factors affecting PICU admission and length of ward stay, we used the multivariate regression analysis. Data are presented as mean ± Standard Deviation (SD), and median (Inter Quartile Ranges (IQR)) for continuous data and number and percentage for categorical data. *p* value < 0.05 is considered significant.

## 3 RESULTS

We initially screened 262 cases diagnosed as acute bronchiolitis and only 194 were included in the study, Fig. (**1**) shows the inclusion and grouping of our cases.

Out of the 194 cases included in the study, 129 (66.49%) were males. The mean age was 3.3 ± 1.52 months, median 3 (2-4) months. The mean length of ward stay was 4.15 ± 1.82 days and the median was 4 (3-5) days.

Age, sex, presence of poor feeding, poor activity, retractions, highest respiratory rate, grunting, crackles, presence of fever on admission, lowest oxygen saturation, lowest Ph, development of cyanosis, development of apnea, ward Length Of Stay (LOS), and X-ray category were entered into analysis.

File reviews showed that 187 (96.39%) of our cases admitted for acute bronchiolitis had X-ray done in the Pediatric Emergency Room (ER) which is a unit within the Pediatric Department at Al-Adan hospital. During inpatient admission, 93 cases (47.9%) did not have X-ray during their admission course (control group), while in 60 patients (30.9%) one X-ray was ordered (group one), and in 41 cases (21.1%) two or more X-rays were done (group 2).

Table **1** compares the characteristics of our cases according to their groups. It shows that there is no statistical significance between the three groups as regards age, gender and history of fever.

The same Table **2** shows that significantly more severe cases of bronchiolitis were subjected to two or more X ray during their admission. Additionally, patients in group 2 were more likely to be prescribed antibiotics, have longer admissions, and required PICU admission compared to the patients in group one and the controls.

Among the cases in groups one and two, in 56.25% of cases the doctor documented the reason for ordering the X ray, and those were; 30.8% desaturation, 24.4% development of apnea, 28.2% possible aspiration, 16.6% miscellaneous reasons (development of new fever, worsening cough, marked distress).

Regarding the results of the performed X-rays, more than 75% of cases showed the typical picture of hyperinflation which was the same finding reported in the admission X-Ray. Among the other cases, increased bronchiolar infiltrate was reported in 15 cases, and 8 patients showed lung collapse (6 were right upper lobe lung collapse).

Upon finding that more cases in group 2 needed PICU admission, we performed a logistic regression analysis to evaluate the possible role of ordering X-rays with regard to the need for PICU admission. After controlling for different confounding factors, Table **2** shows the significant predictors for the need to PICU admission among moderate and severe cases of acute bronchiolitis. Respiratory distress, pH, oxygen saturation and development of apnea were all reported. X-ray category was not detrimental for PICU admission and clinical severity parameters were the indicators for PICU need.

We further examined cases who did not require PICU admission for the factors affecting their ward length of stay. Table **3** shows that after correction of different clinical parameters, requesting X-rays during hospital admission was associated with increased Length Of Stay (LOS).

## 4 DISCUSSION

Nearly 50% of the documented reasons for ordering an X ray in our series of bronchiolitis patients are simply clinical severity symptoms and signs. Additionally, these severity symptoms and signs are significant predictors for the need to PICU admission among our series of acute bronchiolitis cases. These results agree with Ochoa Sangrador *et al.* [7], who could not recommend any of the symptom or severity scores for application in clinical practice, whether for their predictive value or as prognostic tools. Therefore, using either as a reason for imaging can be questionable.

The results of the current study also revealed that in 28.8% the reason behind repeated imaging was possible aspiration as documented by doctors. The imaging procedure in this sector of patients can be accounted for according to Barnes *et al.* [8]. The latter authors mentioned that diffuse bronchiolar disease likely represents an under-recognized form of aspiration-related lung disease and radiological features associated with this disorder are distinctively different from those seen in aspiration pneumonia. In 2015 Hu *et al.* [9], also emphasized that subjects with recognizable predisposing factors for aspiration and who report a history of recurrent pneumonia are at increased risk for diffuse aspiration bronchiolitis with its characteristic imaging features.

The current study also revealed that significantly more patients from those subjected to two or more X-rays were prescribed antibiotics, which agrees with the fears of Suchuh *et al.* [5]. The latter authors reported that imaging abnormalities compatible with uncomplicated bronchiolitis often prompt physicians to prescribe antibiotics. Surprisingly, Mittal *et al.* [10], demonstrated a significant decrease in X ray utilization but not in antibiotics use after inpatient bronchiolitis guideline implementation which might be a further warning sign that such malpractice is now unleashed even after eliminating the triggering factors.

In the logistic regression analysis, the number of X rays was significantly contributing to the LOS for the cases of acute bronchiolitis in the current study. Worth mentioning here is that Mittal *et al.* [10], emphasized the role of inpatient bronchiolitis guideline implementation, with the decrease in X ray utilization, in decreasing the LOS. Additionally, in 2005 Christakis *et al.* [11], reported that the factors associated with the greatest increases in LOS in their cases of bronchiolitis included higher severity scores and use of antibiotics, bronchodilators, and corticosteroids. According to Escorihuela Esteban *et al.* [12], and Piñero Fernández *et al.* [13], LOS was longer in the group of bronchiolitis patients receiving antibiotics.

It is worth mentioning that, although this is the first study to explore imaging utilization among cases with moderate to severe bronchiolitis in our hospital, it has its own limitations. Being a pilot study we didn’t have previous data to do power analysis and calculate the required number needed to get a significant change in length of stay. A high percentage of our patients didn’t have a reason for the requested X-rays documented in the files that might have affected the results.

## CONCLUSION

In conclusion, the current study shows that in more than 40% of the studied bronchiolitis cases the reason for X ray is unaccounted for and it is a clinical severity item in nearly half of cases. We recommend strict adherence to the acute bronchiolitis guidelines to decrease the X ray utilization and subsequent unnecessary antibiotic use aiming at decreasing the LOS and its costs. Additionally, hospital audits should be routinely done to encourage doctors for better documentation of the reasons imaging studies are ordered, whether in the ER room or in the wards.

## Figures and Tables

**Fig. (1):**
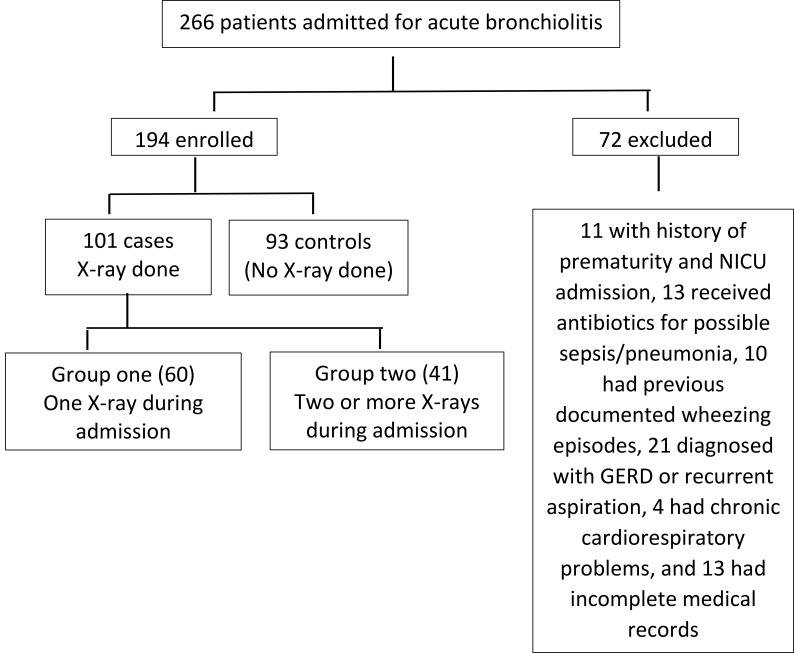
classification of cases with acute bronchiolitis based on X-ray requirement during admission.

**Table 1:** Comparison of the studies cases according to number of X-rays done during admission.

-	**Number of X Rays During Admission**			*p*
-	Control 93 (47.9%)	Group One 60 (30.9%)	Group Two 41 (21.1%)	-
Male	64 (49.6%)	39 (30.2%)	26 (20.2%)	0.795^&^
Age (months)	3.39 ± 1.72 3 (2-4.5)	3.4 ± 1.47 3 (2.5-4)	2.96 ± 1.02 2.5 (2.25-3.5)	0.978 (1vs2) * 0.102 (2vs3) 0.14 (1vs3)
Fever	10 (47.6%)	7 (33.3%)	4 (19%)	0.965^&^
**Acute bronchiolitis severity**				
Moderate	84 (51.2%)	53 (32.3%)	27 (16.5%)	0.001^&^
severe	9 (30%)	7 (23.3%)	14 (46.7%)	
Developed apnea	8 (25%)	10 (31.2%)	14 (43.8%)	0.001^&^
Developed Cyanosis	14 (33.3%)	13 (31%)	15 (35.7%)	0.016^&^
O2 saturation	93.57 ±4.154 94.5 (91.25-97.0)	92.67±5.33 94 (90-96.75)	92.88±5.35 95 (90.5-96.5)	0.246(1vs2) * 0.846(2vs3) 0.423 (1vs3)
pH lowest	7.33±0.056 7.32 (7.3-7.37)	7.32±0.045 7.33 (7.29-7.35)	7.32±0.05 7.32 (7.29-7.36)	0.292(1vs2) * 0.672(2vs3) 0.642 (1vs3)
Ward LOS	3.85±1.66 4 (3-4.5)	4.12±1.48 4 (3-4.75)	4.88±2.4 4 (3-6)	.313(1vs2) * 0.052 (2vs3) 0.005 (1vs3)
Admitted to PICU	5 (26.3%)	6 (31.6%)	8 (42.1%)	0.040^&^
Antibiotics added after admission	17 (32.1%)	19 (35.8%)	17 (32.1%)	0.016^&^

Categorical data presented as numbers (%), continuous data presented as Mean ± SD (median [25^th^ -75^th^ percentiles]), * Student t-test, ^&^Chi-square test/Fishes exact test as appropriate.

**Table 2:** The predictors for the need of PICU admission among moderate and severe cases of acute bronchiolitis.

	Sig	Exp B	95% CI	
Lowest Oxygen saturation	0.028	.883	0.79	0.987
Highest respiratory rate	0.01	1.099	1.023	1.181
Lowest pH	0.016	0.000	0.000	0.058
Development of apnea	0.001	9.67	2.511	37.4
Poor activity	0.055	3.59	0.972	13.28

**Table 3:** The factors affecting the hospital length of stay for cases with acute bronchiolitis not needing PICU admission.

	B	Sig	95% CI	
Total number of x-rays	0.746	< 0.001	0.441	1.050

Age, sex, presence of poor feeding, poor activity, retractions, highest respiratory rate, grunting, crackles, presence of fever on admission, lowest oxygen sat, lowest Ph, development of cyanosis, development of apnea, and X-ray category were entered into analysis.
